# Doublon dynamics and polar molecule production in an optical lattice

**DOI:** 10.1038/ncomms11279

**Published:** 2016-04-14

**Authors:** Jacob P. Covey, Steven A. Moses, Martin Gärttner, Arghavan Safavi-Naini, Matthew T. Miecnikowski, Zhengkun Fu, Johannes Schachenmayer, Paul S. Julienne, Ana Maria Rey, Deborah S. Jin, Jun Ye

**Affiliations:** 1 JILA, National Institute of Standards and Technology and University of Colorado, Boulder, Colorado 80309, USA; 2 Department of Physics, University of Colorado, Boulder, Colorado 80309, USA; 3 Joint Quantum Institute, University of Maryland and National Institute of Standards and Technology, College Park, Maryland 20702, USA

## Abstract

Polar molecules in an optical lattice provide a versatile platform to study quantum many-body dynamics. Here we use such a system to prepare a density distribution where lattice sites are either empty or occupied by a doublon composed of an interacting Bose-Fermi pair. By letting this out-of-equilibrium system evolve from a well-defined, but disordered, initial condition, we observe clear effects on pairing that arise from inter-species interactions, a higher partial-wave Feshbach resonance and excited Bloch-band population. These observations facilitate a detailed understanding of molecule formation in the lattice. Moreover, the interplay of tunnelling and interaction of fermions and bosons provides a controllable platform to study Bose-Fermi Hubbard dynamics. Additionally, we can probe the distribution of the atomic gases in the lattice by measuring the inelastic loss of doublons. These techniques realize tools that are generically applicable to studying the complex dynamics of atomic mixtures in optical lattices.

## Abstract (web summary)

The ability to produce polar molecules in an optical lattice is used to prepare a system where occupied lattice sites contain a Bose-Fermi doublon. Here, the authors apply this approach to optimize the polar molecule creation process and to study complex out-of-equilibrium Bose-Fermi-Hubbard dynamics.

## Graphical abstract

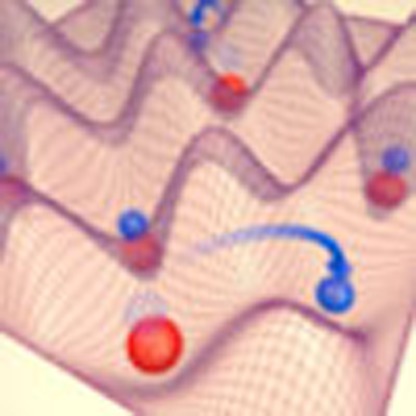

Polar molecules with long-ranged dipolar interactions are ideally suited to the exploration of strongly correlated quantum matter and intriguing phenomena such as quantum magnetism, exotic superfluidity and topological phases^1^,^2^,^3^,^4^,^5^,^6^,^7^,^8^. The recent observation of the dipole-mediated spin-exchange interaction in an optical lattice^9^ and the demonstration of the many-body nature of the spin-exchange dynamics^10^ mark important steps for the use of polar molecules to study strongly correlated matter. While this initial work was done with a molecular filling fraction of only ∼5% in a three-dimensional (3D) lattice^9^, more recent work has demonstrated a quantum synthesis scheme for molecule production in the lattice that relies on careful preparation of the initial atomic gases^11^. This led to a reduction in the final entropy of polar molecules by a factor of ∼4, and, correspondingly, a much higher filling fraction of ∼25% that opens up the possibility for studying non-equilibrium, many-body spin dynamics in a quantum gas of polar molecules where every molecule is connected to others.

The quantum synthesis approach reported in ref. 11 starts by preparing atomic insulator states that depend on atomic interactions, quantum statistics and low temperature^12^. However, realizing the full potential of this approach requires not only control over the atomic distributions, but also a detailed understanding of the molecule creation process.

Here we investigate this important step by leveraging our capability of molecule production in an optical lattice to create a clean system of doublons^13^. This technique allows us to additionally study 3D Bose-Fermi Hubbard dynamics. After creating ground-state molecules, we efficiently remove all unpaired atoms from the lattice and convert the molecules back to free atoms (in their lowest hyperfine states of 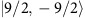 for ^40^K and 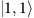 for ^87^Rb, where 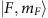 denotes the hyperfine state and its projection onto the magnetic field). This realizes a lattice where the sites are either empty or occupied by individual doublons that comprise a pair of bosonic and fermionic atoms. This well-defined initial state allows us to directly address limitations in the molecule creation process by probing the efficiency with which these doublons are converted back to molecules under various experimental conditions that affect atomic tunnelling rates, higher Bloch-band populations and the adiabaticity of a magnetic-field sweep through a higher partial-wave Feshbach resonance. Furthermore, this well-initialized, non-equilibrium state of a disordered doublon distribution provides an ideal platform to explore the many-body dynamics of a lattice-confined Bose-Fermi mixture in a regime that is beyond the current simulation capabilities.

## Results

### Preparing the doublons

**Figure 1:**
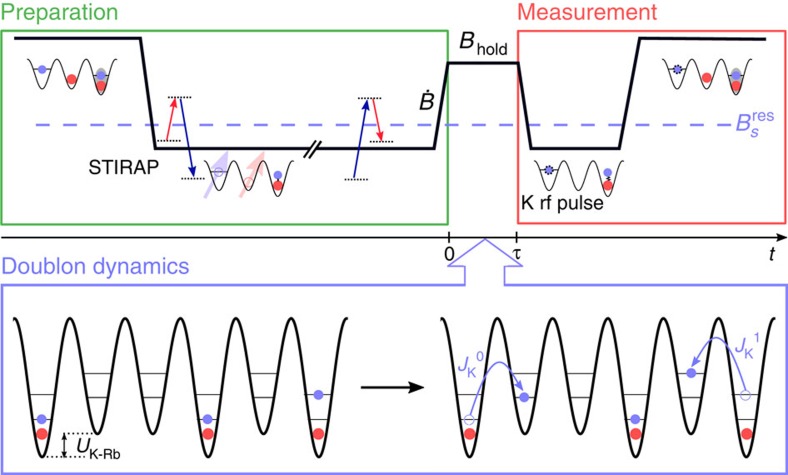
A schematic of the experiment. Starting with a mixture of K, Rb and doublons (the smaller blue ball, the larger red ball and the pair grouped with grey background, respectively) in a 3D lattice, we sweep the magnetic field from above the *s*-wave Feshbach resonance (at 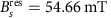) to below the resonance to create Feshbach molecules. These molecules are then transferred to their ro-vibrational ground state via STIRAP (stimulated Raman adiabatic passage). After unpaired atoms are removed with resonant light, the STIRAP process is reversed to transfer the ground-state molecules back to Feshbach molecules. The field is then swept above 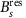 to dissociate the molecules and create doublons. After holding for a time, 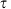, at *B*_hold_, we measure the conversion efficiency when sweeping the field below 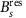 to re-form Feshbach molecules. To detect molecules, we use a rf pulse to spin flip the unpaired K atoms to a dark state (ball with black dashed edge) before dissociating the Feshbach molecules and imaging K atoms. The bottom panel illustrates possible dynamics of the doublons during *B*_hold_. As shown schematically, lattice sites populated with a K and a Rb atom have an interaction energy shift 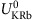. The K tunnelling energies in the lowest and first excited bands are denoted by 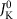 and 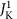, respectively. Rb tunnelling happens at a slower rate since it experiences a deeper lattice.

The experiment proceeds in steps as depicted schematically in Fig. 1. To prepare the doublons, we create a sample of molecules in their ro-vibrational ground state in the lattice as described in ref. 11 and then remove unpaired atoms with resonant light, so that all lattice sites are either empty or contain a single molecule. We then transfer the ground-state molecules back to a weakly bound Feshbach molecule state, followed by a magnetic-field (*B*) sweep to above the resonance to create a clean system of doublons. The solid black line in the upper panel of Fig. 1 shows schematically *B* relative to the *s*-wave Feshbach resonance (dashed line) that is used to manipulate the atomic inter-species interactions and to create molecules. After this preparation, the doublons are left to evolve in the lattice for a variable time 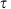. Our measurement then consists of sweeping *B* to below the resonance to associate atoms into Feshbach molecules and determining the fraction of K atoms that form molecules. Specifically, we measure the molecule number using the following protocol. We first apply radio frequency (rf) to spin-flip the unpaired K atoms to another hyperfine state, which renders the unpaired K atoms invisible for subsequent molecular detection. We then sweep *B* back above the resonance to dissociate the molecules, and measure the number of resulting K atoms by spin-selective resonant absorption imaging. The conversion efficiency is determined by dividing this molecule number by the total number of K atoms measured when we do not apply the rf.

### *d*-wave Feshbach resonance

**Figure 2:**
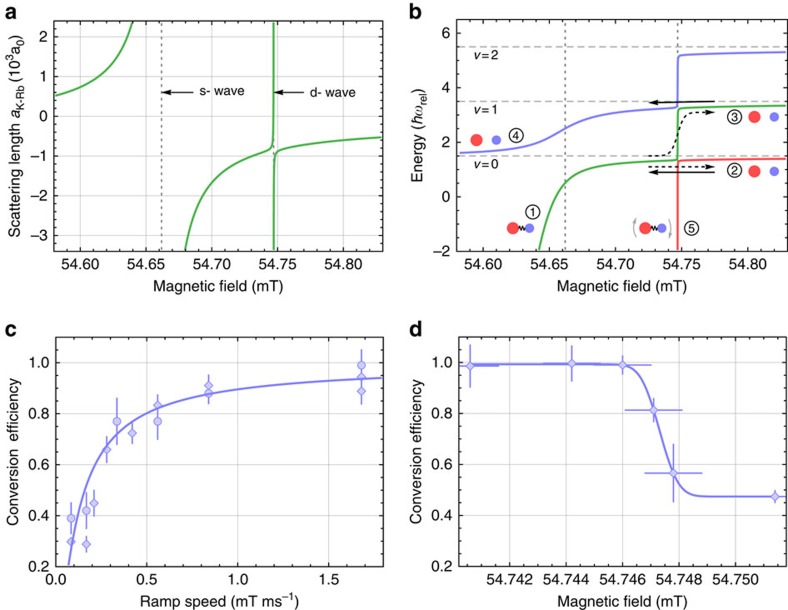
The *d*-wave Feshbach resonance. (**a**) The theoretical K-Rb scattering length, *a*_K-Rb_, is shown as a function of the magnetic field for the broad *s*-wave Feshbach resonance and a narrow *d*-wave resonance, based on the formula and parameter values described in Methods. (**b**) Crossing the *d*-wave resonance affects the pair states for K and Rb. Dashed and solid arrows show the effect of the variable rate sweep that creates doublons and the subsequent fast magneto-association sweep, respectively. Dashed vertical lines mark the positions of the Feshbach resonances. (**c**) Measurement of molecule conversion efficiency at 35 *E*_R_ (circles) and 30 *E*_R_ (diamonds), with the latter data exponentiated by (35/30)^3/4^=1.12 to account for the expected dependence on lattice depth. The solid curve shows a fit to a Landau-Zener probability P (see text), which gives a resonance width of 9.3(7) × 10^−4^ mT. (**d**) The magnetic field at which this resonance occurs is determined by sweeping up to various fields at 0.018 mT ms^−1^, then sweeping down at 0.18 mT ms^−1^. The position of the resonance extracted from this measurement at *V*_latt_=35*E*_R_ is 54.747(1) mT. All error bars represent 1−*σ* standard error.

We begin by investigating a narrow *d*-wave Feshbach resonance^14^,^15^,^16^,^17^ that is located less than 0.1 mT above the 0.3-mT-wide *s*-wave resonance that is used for making molecules (Fig. 2a). With a pair of atoms confined in the same lattice site, the on-site density is orders of magnitude higher than that in ordinary optical traps, and thus this narrow Feshbach resonance can adversely affect the magneto-association process, where *B* is swept down from above the *s*-wave resonance to create molecules. Crossing the *d*-wave resonance too slowly will produce *d*-wave molecules, which will not be coupled to the ground state by the subsequent STIRAP laser pulses, as the process is weak and off resonance. If *B* is swept sufficiently fast to be diabatic for this narrow resonance (but still slow enough to be adiabatic for the broad *s*-wave resonance), crossing the *d*-wave resonance has no impact; however, the high effective densities at each site in an optical lattice can make it challenging to sweep fast enough. Although we study here specific resonances for the K-Rb system, the possibility of having to cross other Feshbach resonances and the issue of sweep speeds are general to magneto-association of atoms in an optical lattice.

In the experiment illustrated in Fig. 1, we investigate the *d*-wave resonance by varying the rate, 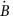, and the final value, *B*_hold_, of the sweep that creates doublons. We then measure the subsequent molecule conversion efficiency after 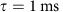 using a fast 1.68-mT ms^−1^ magneto-association sweep. Figure 2b illustrates relevant states, above and below the resonance, for two atoms in a lattice site^18^,^19^: at low fields these are the *s*-wave molecule (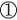), *d*-wave molecule (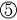) and unbound atoms (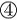), and at high fields these are unbound atoms in the ground band of the lattice (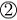) and atoms with a band excitation in their relative motion (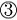). For simplicity, we illustrate states for a harmonic potential whose trap frequency 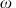 is the same for both atoms, with eigenstates of relative motion denoted by 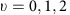. The dashed arrows show the diabatic (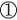
→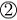) and adiabatic (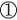
→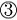) trajectories for the dissociation of *s*-wave Feshbach molecules when crossing the *d*-wave resonance, while the solid arrows show the diabatic trajectories (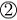
→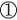 and 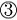
→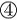) for the subsequent, fast magneto-association sweep.

Figure 2c shows the measured molecule conversion efficiency as a function of 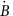 when sweeping across the *d*-wave resonance from 54.56 to 56.24 mT (there are no other resonances in this field range), while Fig. 2d shows the effect of the final field *B* for a relatively slow, 0.018-mT ms^−1^, sweep. The data are taken for lattice depths of *V*_latt_=35*E*_R_ (circles) and 30*E*_R_ (diamonds), where *E*_R_=*ħ*^2^*k*^2^/(2*m*_Rb_) is the recoil energy for Rb, *m*_Rb_ is the Rb atom mass, 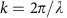 and 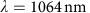. For our highest sweep rates, or when *B*_hold_ is below the *d*-wave resonance, the measured molecule conversion efficiency is near unity. This high conversion of doublons^20^,^21^,^22^ is crucial for the quantum synthesis approach to producing molecules with a high filling fraction in the lattice. The near-unity conversion also provides an excellent starting point for diagnosing potential limitations to molecule production, and the data in Fig. 2c,d clearly show the negative effect that the *d*-wave resonance can have on magneto-association in the lattice.

The lines in Fig. 2c,d show fits used to extract the width (Δ_*d*_) and position of the *d*-wave resonance. We use a Landau-Zener formalism^23^ where the probability to cross the *d*-wave resonance diabatically, and therefore create *s*-wave Feshbach molecules in the subsequent magneto-association step, is 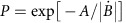, where *A* depends on the on-site densities and the Feshbach resonance parameters. By approximating the sites in the deep optical lattice as harmonic oscillator potentials, we extract Δ_*d*_ using 
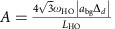
, where 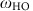 is the harmonic trap frequency for relative motion of the two atoms (see Methods) and 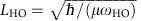 is the harmonic oscillator length with the doublon reduced mass 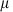 (ref. 24) (we note that the right-hand side of equation (26) in this reference is missing a factor of 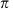). From an exponential fit (line in Fig. 2c), *A*=0.110(7)* *mT* *ms^−1^, and using a background scattering length of *a*_bg_=−187(5)*a*_0_ (ref. 25), where *a*_0_ is the Bohr radius, we extract a width of 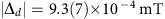. By fitting an error function (line) to the data in Fig. 2d, we determine the location of the resonance to be 54.747(1)* *mT, which is consistent with previous experiments where atom loss was observed^14^,^16^.

The precise determination of the width of the *d*-wave resonance allows us to gauge its significance in molecule creation. Our typical sweep rate of 0.34 mT* *ms^−1^ for magneto-association, which has remained the same since the first creation of KRb molecules in an optical lattice^22^, gives ∼70% probability of being diabatic when crossing the *d*-wave resonance. This suggests that we create a substantial fraction of *d*-wave molecules that are dark to our detection (in Fig. 2b). These *d*-wave molecules may have played a role in limiting the lattice filling fraction for polar molecules achieved in ref. 11.

### Short-time tunnelling dynamics

Tunnelling dynamics of doublons in the lattice^26^ can also affect molecule production. In the quantum synthesis approach, achieving a high lattice filling for molecules requires not only the preparation of a large fraction of lattice sites that have doublons, but also that these doublons are not lost due to tunnelling and/or collisions prior to conversion to molecules. In our system, K feels a lattice depth that, in units of recoil energy, is 2.6 times weaker than for Rb due to differences in atomic mass and polarizability. Consequently, K tunnels faster than Rb. While a sufficiently deep lattice can prevent tunnelling of both K and Rb, practically this may not be possible in all cases, especially for polar molecule production using two atomic species that have large differences in mass and polarizability.

**Figure 3:**
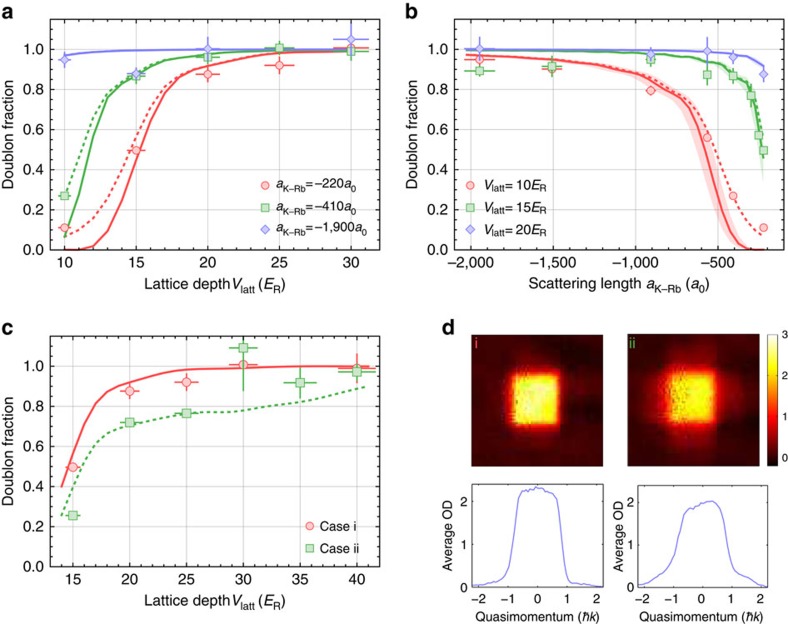
Interaction and tunnelling dynamics of doublons in the lattice. (**a**) The remaining doublon fraction is shown for three scattering lengths as a function of the lattice depth. (**b**) The doublon fraction is plotted for three lattice depths as a function of the scattering length. (**c**) The doublon fraction for 1.68 mT ms^−1^ sweeps, 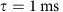 and *a*_K-Rb_=−220*a*_0_ is shown as a function of the lattice depth for the case of higher excited-band fraction (squares) and lower excited-band fraction (circles). (**d**) Band-mapping images of the initial K gas are shown for the two different initial temperatures, where image i corresponds to the red circle data points and ii corresponds to the green square data points in (**c**). Each image is the average of three measurements. The colour bar indicates the optical depth (OD). Below the images, we display the OD for a horizontal trace through the image, with averaging from −*ħk* to +*ħk* in the vertical direction. All error bars represent 1−*σ* standard error.

Figure 3 illustrates doublon dynamics due to the interplay between tunnelling and interactions, which we control by varying the lattice depth, interspecies scattering length *a*_K-Rb_ and band population. The fraction of doublons that remain after 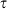 is essentially equal to the measured molecule conversion efficiency described above. We note that for *a*_K-Rb_>−850*a*_0_, the *B* sweep crosses the *d*-wave Feshbach resonance with a 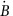 that varies from 0.5 to 1.9 mT ms^−1^. Using our measured width of the *d*-wave resonance, the data presented in Fig. 3 have been multiplied by a factor that increases the doublon fraction to account for the finite 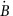 when crossing the *d*-wave resonance. Figure 3a shows the effect of the lattice depth for 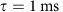 at three different values of *B*_hold_, corresponding to different values of *a*_K-Rb_. This timescale is relevant for both molecule production and K tunnelling dynamics. We observe that the remaining doublon fraction is highly sensitive to the lattice depth for weak interspecies interactions, for example, *a*_K-Rb_=−220*a*_0_, with a lower doublon fraction for shallower lattices that exhibit higher tunnelling rates. For stronger interactions, the dependence on lattice depth becomes less significant and almost disappears in the strongly interacting regime, for example, *a*_K-Rb_=−1,900*a*_0_. Similar behaviour is observed if we fix the lattice depth but vary the interspecies interactions, as shown in Fig. 3b.

### Modelling

The data in Fig. 3 clearly show evidence of decay of doublons due to tunnelling that is affected by both the lattice depth and interspecies interactions. We can model these doublon dynamics with the following Hamiltonian:


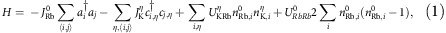


where *η*=0 and 1 denote, respectively, the ground and the first excited lattice bands. The first and second terms are the kinetic energy of the K and Rb atoms, respectively. Here, 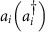 is the bosonic annihilation (creation) operator for a Rb atom at lattice site *i* in the lowest band, and 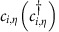 is the fermionic annihilation (creation) operator for a K atom at lattice site *i* and band 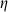. We use 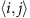 to indicate nearest-neighbour hopping between sites *i* and *j* with matrix element 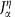 with 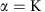 or Rb. The third term describes the inter-species on-site interactions with matrix element 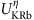. The last term is the on-site intra-species interaction between ground-band Rb atoms with strength 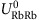, with 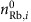 as the occupation of site *i*.

The tunnelling rates and interaction energies are calculated for a particular *V*_latt_ and *a*_K-Rb_ (ref. 27). For example, for *V*_latt_=10*E*_R_, 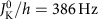, 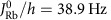. The solid curves in Fig. 3a,b show the calculations based on the Hamiltonian given in equation (1), where we have neglected Rb tunnelling by setting 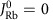. We start with a single doublon, evolve the K for a hold time 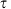, and then extract the doublon fraction from the probability that the K atom remains on the same site as the Rb atom. In this treatment, we ignore the role of the magnetic-field sweeps. Calculations for a single doublon (solid lines), where the initial decay scales as 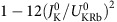, agree well with the data, except at doublon fractions below ∼30%, where the disagreement arises from the finite probability in the experiment that a K atom finds a different Rb partner. Simulating a Gaussian distribution of doublons with 10% peak filling accounts for this effect (dashed lines) (see Methods). The good agreement of these calculations with the data shows that tunnelling of K, which is suppressed for deeper lattices, is the dominant mechanism for the reduction of the doublon fraction at short (∼1 ms) times. The on-site interaction with Rb suppresses the K tunnelling when the interaction energy becomes larger than the width of the K Bloch band^28^.

When studying doublon dynamics measured for two different initial atom conditions, we find indirect evidence for excited-band molecules. Here, we compare results for our usual molecule preparation using atomic insulators to a case where we start with a hotter initial atom gas mixture at a temperature above that for the Rb Bose-Einstein condensation transition. Using a band-mapping technique, we measure the initial population of K in the ground and first excited band, as shown in Fig. 3di and ii (see Methods). We find that 11(2)% of the K atoms occupy the first excited band for the colder initial atom gas (these conditions are similar to those in ref. 11 and are used in all the measurements described in this work, except for the green squares in Fig. 3c). When starting with the hotter atom gas, we measure a significantly higher K excited-band population of 31(6)%. When looking at doublon dynamics for these two cases (Fig. 3c), we observe a lower doublon fraction for the hotter initial gas for *V*_latt_≤25*E*_R_. These data are taken for 1.68 mT ms^−1^ sweeps, 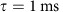 and *a*_K-Rb_=−220*a*_0_.

The lower doublon fraction can be explained by excited-band K atoms, which have a high tunnelling rate (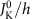 and 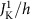 are 89.3 and 1110 Hz, respectively, for *V*_latt_=25*E*_R_). The presence of excited-band K atoms suggests that the *B* sweeps for magneto-association (and dissociation) couple excited-band K atoms (plus a ground-band Rb atom) to excited-band Feshbach molecules. Moreover, the data suggest that the conversion efficiency for the excited-band Feshbach molecules is still high for *V*_latt_≤25*E*_R_ since the observed difference in the initial excited-band K atoms is similar to the observed difference (roughly 20%) in the doublon fraction (Fig. 3c). Since, in our preparation scheme, the doublons are directly formed from the dissociation of ro-vibrational ground-state molecules, these results further indicate that a polar molecule sample prepared from a finite-temperature atom gas can contain a small fraction of molecules in an excited motional state in the lattice. We also observed a Rb excited-band population of 31(5)% after loading the thermal gas in the lattice; however, even for the excited band, the off-resonant Rb tunnelling is slow compared to the 1-ms time scale of the measurements presented in Fig. 3.

The green dashed curve in Fig. 3c shows the theoretical results for a K excited-band fraction of 24%. For comparison, the red solid curve, which is the same as the red curve in Fig. 3a, includes no excited-band population. The estimated excited band fraction ignores the effects of harmonic confinement on tunnelling, which are more significant for the hotter initial atom gas, where the resulting molecular cloud is also larger. For the hotter initial atom gas, the green dashed curve overlaps the data at the shallower lattice depths, but deviates from the measured doublon fraction at larger lattice depths (the excited band fraction of the initial K gas is independent of lattice depth). This may be expected since in the limit of a very deep lattice and a fully adiabatic magneto-association sweep, one expects that only the heavier atom (Rb) in excited bands (plus a ground-band K atom) will couple to excited-band Feshbach molecules. Future studies of the magneto-association process in a lattice for systems such as K-Rb where centre-of-mass and relative motion are coupled^29^ would be interesting and relevant to polar molecule preparation.

### Long-time tunnelling dynamics

**Figure 4:**
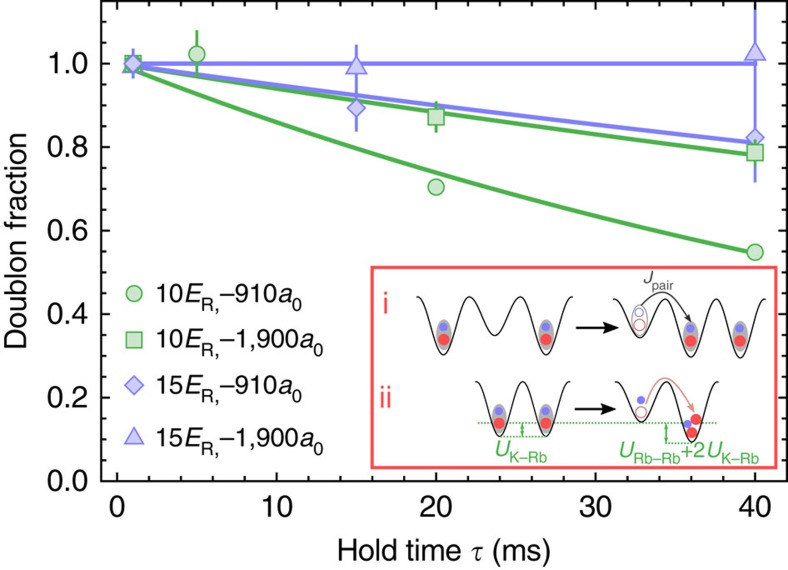
Longer time dynamics. The dependence of the doublon fraction on the hold time 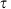 in the lattice for both 10*E*_R_ (green circles and squares) and 15*E*_R_ (blue diamonds and triangles) for either *a*_K-Rb_=−1,900*a*_0_ (circles, diamonds) or *a*_K-Rb_=−1,900*a*_0_ (squares, triangles). The lines are fits to an exponential decay and are intended only as guides to the eye. (Inset) The doublon decay can involve tunnelling of doublons through empty sites (i) prior to loss by the Rb tunnelling process illustrated in (ii). All error bars represent 1−*σ* standard error.

In Fig. 4, we present data taken for 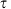 up to 40 ms, in order to look for the effects of Rb tunnelling. Measurements of the remaining doublon fraction are shown for two lattice depths (10*E*_R_ and 15*E*_R_) and two values of *a*_K-Rb_ (−910*a*_0_ and −1900*a*_0_). In Fig. 4, the doublon fraction has been normalized by the measured value for 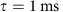 in order to remove the effect of the shorter-time dynamics that are presented in Fig. 3a,b. Similar to the shorter-time dynamics, at the longer hold times we observe a reduction in the doublon fraction that is suppressed for a deeper lattice and for strong inter-species interactions. Modeling these dynamics is theoretically challenging, and the lines in Fig. 4 are exponential fits that are intended only as guides to the eye. Compared to doublons composed of identical bosons^13^ or fermions in two-spin states^30^, the heteronuclear system has the additional complexities of two particle masses, two tunnelling rates and two relevant interaction energies. For example, for large *a*_K-Rb_, the interspecies interactions will strongly suppress Rb tunnelling from a doublon to a neighbouring empty site. Similarly, tunnelling of a doublon to an empty lattice site is a slow second-order process at the rate 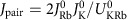 due to the energy gap of 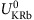 (Fig. 4 inset i). However, Rb tunnelling between two neighbouring doublons, which creates a triplon (Rb-Rb-K) on one site and a lone K atom on the other site (Fig. 4 inset ii), may occur on a faster time scale due to a much smaller energy gap of 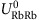, which is smaller than the K tunnelling bandwidth. While the theoretical description is complicated, we observe that the time scale of the doublon decay roughly matches 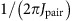.

### Measuring atomic distributions with doublon detection

The studies discussed thus far demonstrate that the Feshbach molecule conversion that we use to detect doublons could potentially underestimate the doublon fraction. For example, the conversion efficiency of doublons containing excited-band atoms is complicated to calculate and is likely to be less than 1. In addition, the efficiency of converting doublons to Feshbach molecules depends on the magnetic-field sweep rate, and, as shown in Fig. 2c, a very slow sweep does not always yield a unity conversion efficiency. Finally, Feshbach molecules can suffer losses from inelastic collisions with other Feshbach molecules or unpaired K atoms^31^, which could reduce the measured number. Given these factors and the importance of measuring the doublon fraction as a powerful diagnostic for optimizing molecule production from ultracold atoms in a lattice, we have implemented a second, complementary approach for measuring the doublon fraction using inelastic collisional loss in the initial atomic mixture, without the molecular purification step. In our system, inelastic collisions are initiated by transferring the Rb atoms from the 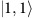 to the 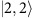 state. Collisions of the 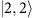 Rb atoms with K can result in spin relaxation back to the Rb *F*=1 manifold. At *B*=55 mT, the 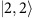 state is higher in energy by *h* × 8.1 GHz; this inelastic collision releases a large amount of energy compared to the trap depth and therefore results in atom loss from the trap. At a collision energy corresponding to 1 μK, the calculated inelastic collision rate using the coupled channels model of ref. 15 is 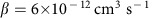, and using the on-site densities in a *V*_latt_=25*E*_R_ lattice, the resulting doublon lifetime is ∼2 ms.

**Figure 5:**
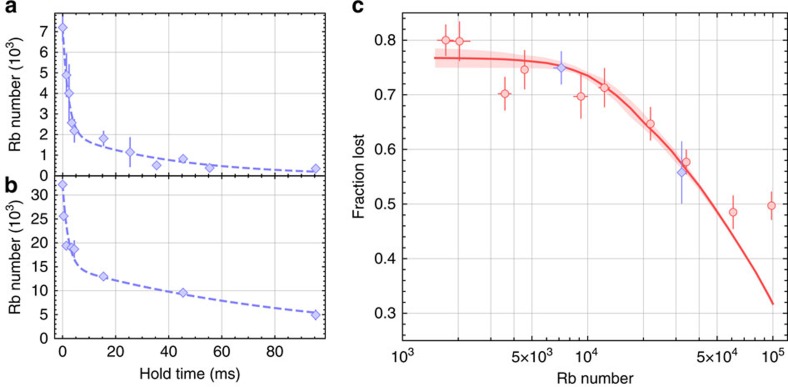
Measuring the initial atomic distributions with spin-changing collisions. (**a**,**b**) Sample data for low and high Rb number, respectively. The fraction of Rb remaining after the fast loss is different between the two cases. (**c**) The fraction of Rb lost after ∼8 ms is plotted as a function of the Rb number in a 25*E*_R_ lattice. The blue diamonds correspond to the data shown in panels (**a**,**b**). At low Rb number, where the Mott insulator has one Rb atom per site, the fraction lost should be equal to the fraction of sites that have a K atom. As the Rb filling increases and the second Mott shell becomes populated, the fraction lost decreases. This technique yields both the filling of K and a measure of Rb atom number that corresponds to the onset of double occupancy of the Rb Mott insulator. All error bars represent 1−*σ* standard error.

In Fig. 5a,b, we show example data for the number of Rb atoms as a function of time after a 2.1-ms rf sweep that transfers Rb atoms to the 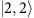 state. We observe a fast loss on the time scale of a few ms, followed by slower loss. We attribute the fast loss to inelastic collisions of Rb atoms in lattice sites shared with K, and the slow loss to tunnelling of atoms followed by inelastic collisions. The dashed lines in Fig. 5a,b show a fit to the sum of two exponential decays with different time constants. We can extract the fraction of Rb that is lost on the short timescale from the fits. We compared this technique with Feshbach molecule formation, and found that the two measurements generally agree.

As a further demonstration of the inelastic collision technique, we use this to probe the initial atomic distribution in the lattice before molecule formation, providing quantitative information on the Rb Mott insulator. Figure 5c shows the fraction of Rb atoms that are lost quickly from a *V*_latt_=25*E*_R_ lattice after the Rb atoms are transferred to the 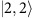 state. For these data, we vary the initial number of Rb atoms that form a Mott insulator in the optical lattice prior to molecule creation. In Fig. 5c, the blue diamonds correspond to the data shown in Fig. 5a,b. For the data shown in circles, the fraction lost is determined by comparing the Rb number measured before to that measured 8 ms after the rf transfer. The solid curve shows a calculation of the expected loss for a Mott insulator with a temperature 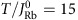 and a total radial harmonic confinement of 33 Hz for Rb. At low Rb number, where we expect only one Rb atom per site in the Mott insulator, the fraction lost is just the K filling fraction, assuming no double occupancy for K. For higher Rb number, double (and eventually triple and higher) occupancy in Mott shells causes a reduction in the fractional loss under the assumption of one Rb and one K lost per inelastic collision. The shaded area indicates a 10% uncertainty in the harmonic trapping frequency and 30% uncertainty in *T*. From the fit, we extract a K filling fraction of 0.77(2), which is in excellent agreement with the measured peak K filling reported in ref. 11. We note the previously measured fraction of Rb converted to Feshbach molecules at low Rb atom number was significantly less, at about 0.5(1) (ref. 11). This disagreement may now be attributed to the large number of K atoms present in the lattice after molecule formation, which can induce losses through inelastic collisions, and to the effect of the *d*-wave resonance when making molecules, as discussed above.

## Discussion

Our investigation of heteronuclear doublons and their conversion to molecules by magneto-association reveals the important roles played by the lattice depth for both atomic species, the inter-species interactions, the population in excited motional states of the lattice and the magnetic-field sweep rate. The doublon dynamics uncovered in this study provides insights into the universal mechanism of their decay and atomic mixture dynamics in a 3D optical lattice, and allows preparation of optimal conditions for producing polar molecules. The highly non-equilibrium state of doublons that we use for these studies also provides an intriguing system for exploring the Hubbard dynamics of a Bose-Fermi mixture, where the behaviour of the many-body system can depend on two different tunnelling rates and two different interaction strengths^28^,^32^. This system sets the stage for performing benchmarking experiments in 1D for theory, and investigating the thermalization of an isolated many-body quantum system, including novel phases such as quasi-crystallization and many-body localization in higher dimensions^33^,^34^,^35^,^36^.

## Methods

### Optical trapping potentials

The preparation of the atomic gas in a 3D lattice, with a wavelength of 1,064 nm, as well as the creation of ground-state polar molecules, follows the procedures described in ref. 11. The lattice is superimposed on a crossed-beam optical dipole trap that is cylindrically symmetric. The dipole trap alone has an axial trap frequency of 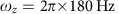 in the vertical direction and a radial trap frequency of 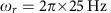 for Rb. The measured optical trap frequencies for K are 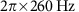 and 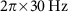.

### Width of the *d*-wave resonance

The scattering lengths reported in Fig. 3 have been calculated using 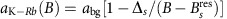 with *a*_bg_=−187(5)*a*_0_, 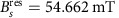 and Δ_*s*_=0.304 mT (ref. 25). Including the *d*-wave resonance, the scattering length can be parameterized by 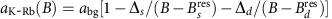 (ref. 37). Using the relation Δ_*d*_<<Δ_*s*_, we can write 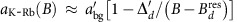 near the *d*-wave resonance, where 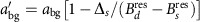 and 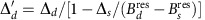. This has the form of an isolated resonance and we can apply the findings of ref. 24, namely that 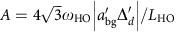, to determine 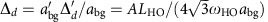. We note that ref. 17 predicts 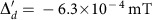, which is larger than our determination of 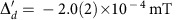.

In this determination, we ignore the coupling between the centre of mass and relative motion that arises from the fact that K and Rb experience different trapping potentials in the optical lattice. We use an effective trap frequency 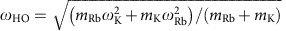 that governs the dynamics in the relative coordinate. Here, *m*_Rb_ and *m*_K_ are the masses of the Rb and K atom, respectively. The trap frequency for Rb is given by 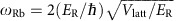, and for the 1,064 nm lattice the trap frequency for K is 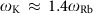. For *V*_latt_=35*E*_R_, 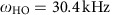.

### Density distribution of doublons

The dashed lines in Fig. 3a,b have been obtained by random sampling of initial doublon positions according to a Gaussian probability distribution of the filling fraction. A peak filling of 10% and widths *σ*_*x*_=*σ*_*y*_=6.5*σ*_*z*_=21 sites have been used, corresponding to *N*≈2,000 sites occupied with a doublon. The experimentally determined cloud sizes are slightly larger (*σ*_*x*_=25-42 sites), but we confirmed that the resulting doublon fraction is converged with respect to the cloud size. *In-situ* absorption images of the cloud are consistent with a Gaussian distribution of 5–10% peak filling. Tunnelling of Rb is neglected in the model, where initially each K atom is localized on a site containing a Rb atom and the doublon fraction is defined as the probability to find the K atom on a site with Rb after the evolution time 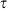.

### Band mapping

To measure the excited-band fraction of the initial K atoms, we use a band-mapping technique (Fig. 3d). Starting with the K atoms in the 3D lattice plus optical dipole trap potential, we turn off the lattice in 1 ms and allow the the K gas to expand in the optical dipole trap for a quarter trap period^38^. We image the cloud with a probe beam that propagates along the vertical direction.

## Additional information

**How to cite this article:** Covey, J. P. *et al*. Doublon dynamics and polar molecule production in an optical lattice. *Nat. Commun.* 7:11279 doi: 10.1038/ncomms11279 (2016).

## References

[b1] Baranov M. Theoretical progress in many-body physics with ultracold dipolar gases. Phys. Rep. 464, 71–111 (2008).

[b2] Pupillo G., Micheli A., Büchler H. P. & Zoller P. in Cold Molecules: Theory, Experiment, and Applications (eds Krems R., Friedrich B., Stwalley W. C. CRC Press (2009).

[b3] Carr L. D., DeMille D., Krems R. V. & Ye J. Cold and ultracold molecules: science, technology and applications. New J. Phys. 11, 055049 (2009).

[b4] Lahaye T., Menotti C., Santos L., Lewenstein M. & Pfau T. The physics of dipolar bosonic quantum gases. Rep. Prog. Phys. 72, 126401 (2009).

[b5] Lemeshko M., Krems R. V., Doyle J. M. & Kais S. Manipulation of molecules with electromagnetic fields. Mol. Phys. 111, 1648–1682 (2013).

[b6] Yao N. Y. . Topological flat bands from dipolar spin systems. Phys. Rev. Lett. 109, 266804 (2012). 23368600 10.1103/PhysRevLett.109.266804

[b7] Gorshkov A. V. . Tunable superfluidity and quantum magnetism with ultracold polar molecules. Phys. Rev. Lett. 107, 115301 (2011). 22026682 10.1103/PhysRevLett.107.115301

[b8] Syzranov S. V., Wall M. L., Gurarie V. & Rey A. M. Spin-orbital dynamics in a system of polar molecules. Nat. Commun. 5, 5391 (2014). 25377238 10.1038/ncomms6391

[b9] Yan B. . Observation of dipolar spin-exchange interactions with lattice-confined polar molecules. Nature 501, 521–525 (2013). 24048478 10.1038/nature12483

[b10] Hazzard K. R. A. . Many-body dynamics of dipolar molecules in an optical lattice. Phys. Rev. Lett. 113, 195302 (2014). 25415911 10.1103/PhysRevLett.113.195302

[b11] Moses S. A. . Creation of a low-entropy quantum gas of polar molecules in an optical lattice. Science 350, 659–662 (2015). 26542566 10.1126/science.aac6400

[b12] Safavi-Naini A., Wall M. L. & Rey A. M. Role of interspecies interactions in the preparation of a low-entropy gas of polar molecules in a lattice. Phys. Rev. A 92, 063416 (2015).

[b13] Winkler K. . Repulsively bound atom pairs in an optical lattice. Nature 441, 853–856 (2006). 16778884 10.1038/nature04918

[b14] Zaccanti M. . Control of the interaction in a Fermi-Bose mixture. Phys. Rev. A 74, 041605 (2006).

[b15] Julienne P. S. Ultracold molecules from ultracold atoms: a case study with the KRb molecule. Faraday Discuss. 142, 361–388 (2009). 20151554 10.1039/b820917k

[b16] Bloom R. S., Hu M.-G., Cumby T. D. & Jin D. S. Tests of universal three-body physics in an ultracold Bose-Fermi mixture. Phys. Rev. Lett. 111, 105301 (2013). 25166676 10.1103/PhysRevLett.111.105301

[b17] Ruzic B. P., Greene C. H. & Bohn J. L. Quantum defect theory for high-partial-wave cold collisions. Phys. Rev. A 87, 032706 (2013).

[b18] Busch T., Englert B.-G., Rzewski K. & Wilkens M. Two cold atoms in a harmonic trap. Found. Phys. 28, 549–559 (1998).

[b19] Köhl M., Moritz H., Stöferle T., Günter K. & Esslinger T. Fermionic atoms in a three dimensional optical lattice: observing Fermi surfaces, dynamics, and interactions. Phys. Rev. Lett. 94, 080403 (2005). 15783869 10.1103/PhysRevLett.94.080403

[b20] Thalhammer G. . Long-lived feshbach molecules in a three-dimensional optical lattice. Phys. Rev. Lett. 96, 050402 (2006). 16486906 10.1103/PhysRevLett.96.050402

[b21] Greif D., Tarruell L., Uehlinger T., Jördens R. & Esslinger T. Probing nearest-neighbor correlations of ultracold fermions in an optical lattice. Phys. Rev. Lett. 106, 145302 (2011). 21561200 10.1103/PhysRevLett.106.145302

[b22] Chotia A. . Long-lived dipolar molecules and Feshbach molecules in a 3d optical lattice. Phys. Rev. Lett. 108, 080405 (2012). 22463505 10.1103/PhysRevLett.108.080405

[b23] Mies F. H., Tiesinga E. & Julienne P. S. Manipulation of Feshbach resonances in ultracold atomic collisions using time-dependent magnetic fields. Phys. Rev. A 61, 022721 (2000).

[b24] Julienne P. S., Tiesinga E. & Köhler T. Making cold molecules by time-dependent feshbach resonances. J. Mod. Opt. 51, 1787–1806 (2004).

[b25] Klempt C. . Radio-frequency association of heteronuclear Feshbach molecules. Phys. Rev. A 78, 061602 (2008).

[b26] Jürgensen O., Meinert F., Mark M. J., Nägerl H.-C. & Lühmann D.-S. Observation of density-induced tunneling. Phys. Rev. Lett. 113, 193003 (2014). 25415904 10.1103/PhysRevLett.113.193003

[b27] Greiner M., Mandel O., Esslinger T., Hänsch T. W. & Bloch I. Quantum phase transition from a superfluid to a Mott insulator in a gas of ultracold atoms. Nature 415, 39–44 (2002). 11780110 10.1038/415039a

[b28] Heinze J. . Multiband spectroscopy of ultracold fermions: observation of reduced tunneling in attractive Bose-Fermi mixtures. Phys. Rev. Lett. 107, 135303 (2011). 22026869 10.1103/PhysRevLett.107.135303

[b29] Jachymski K., Idziaszek Z. & Calarco T. Feshbach resonances in a nonseparable trap. Phys. Rev. A 87, 042701 (2013).

[b30] Strohmaier N. . Observation of elastic doublon decay in the Fermi-Hubbard model. Phys. Rev. Lett. 104, 080401 (2010). 20366917 10.1103/PhysRevLett.104.080401

[b31] Ospelkaus S. . Quantum-state controlled chemical reactions of ultracold potassium-rubidium molecules. Science 327, 853–857 (2010). 20150499 10.1126/science.1184121

[b32] Best T. . Role of interactions in ^87^Rb-^40^K Bose-Fermi mixtures in a 3d optical lattice. Phys. Rev. Lett. 102, 030408 (2009). 19257334 10.1103/PhysRevLett.102.030408

[b33] Gopalakrishnan S., Martin I. & Demler E. A. Quantum quasicrystals of spin-orbit-coupled dipolar bosons. Phys. Rev. Lett. 111, 185304 (2013). 24237533 10.1103/PhysRevLett.111.185304

[b34] Martin I., Gopalakrishnan S. & Demler E. A. Weak crystallization theory of metallic alloys. Preprint at http://arxiv.org/abs/1506.03077 (2015).

[b35] Schreiber M. . Observation of many-body localization of interacting fermions in a quasirandom optical lattice. Science 349, 842–845 (2015). 26229112 10.1126/science.aaa7432

[b36] Smith J. . Many-body localization in a quantum simulator with programmable random disorder. Preprint at http://arxiv.org/abs/1508.07026 (2015).

[b37] Jachymski K. & Julienne P. S. Analytical model of overlapping Feshbach resonances. Phys. Rev. A 88, 052701 (2013).

[b38] Murthy P. A. . Matter-wave Fourier optics with a strongly interacting two-dimensional Fermi gas. Phys. Rev. A 90, 043611 (2014).

